# Etiological factors in young patients with Retinal Vein Occlusion

**DOI:** 10.12669/pjms.35.5.546

**Published:** 2019

**Authors:** Serhad Nalcaci, Cumali Degirmenci, Cezmi Akkin, Jale Mentes

**Affiliations:** 1 Dr. Serhad Nalcaci, M.D. Ege University Faculty of Medicine, Department of Ophthalmology, Izmir, Turkey; 2 Dr. Cumali Degirmenci, M.D. Ege University Faculty of Medicine, Department of Ophthalmology, Izmir, Turkey; 3 Prof. Cezmi Akkin, M.D. Ege University Faculty of Medicine, Department of Ophthalmology, Izmir, Turkey; 4 Prof. Jale Mentes, M.D. Ege University Faculty of Medicine, Department of Ophthalmology, Izmir, Turkey

**Keywords:** Etiological factors, Retinal vein occlusion, Young patients

## Abstract

**Objective:**

To present the etiological factors of patients with Retinal Vein Occlusion (RVO) under the age of 50 years.

**Methods:**

The study was conducted at Ege University Medicine Faculty Department of Ophthalmology. The clinical records of patients with RVO under the age of 50 seen between January 2014 and March 2018 were analyzed retrospectively. Forty patients comprised the study. Detailed ophthalmologic examination was performed. Past medical history, drug use, thrombophilic features, hyperviscosity syndromes and pathologies that may cause vasculitis were noted.

**Results:**

Forty patients, 22 (55%) male and 18 (45%) female, were included. Mean age was 41.6 ± 10.01 years. Mean intraocular pressure and best-corrected visual acuity were 16.8 ± 5.47mmHg and 0.76 ± 0.64 logMAR, respectively. Hyperhomocystenemia (15 patients, 37.5%), Behçet’s disease (three patients, 7.5%), diabetes and/or hypertension (16 patients, 40%), methylenetetrahydrofolate reductase gene mutation (11 patients, 27.5%), prothrombin gene mutation (four patients, 10%) and factor V Leiden mutation (five patients, 12.5%) were present among the patients as etiological factor. Multiple etiological factors were detected in 11 (27.5%) patients. Factor V Leiden mutation and methylenetetrahydrofolate reductase gene mutation were detected in one patient (2.5%) with Behçet’s disease. Four patients with diabetes and/or hypertension also had hyperhomocystenemia and one of them had additionally prothrombin gene mutation. Two patients with methylenetetrahydrofolate reductase gene mutation also had a factor V Leiden mutation and one of them had additionally a prothrombin gene mutation. Three patients with methylenetetrahydrofolate reductase gene mutation also had hyperhomocystenemia and one patient with prothrombin gene mutation also had methylenetetrahydrofolate reductase gene mutation.

**Conclusions:**

Etiological factors that might result in RVO in young individuals should be investigated in detail. Targeted therapies may help to prevent development of new RVOs and potential vascular problems in other organs.

## INTRODUCTION

Retinal vein occlusion (RVO) is the second most common retinal vascular disease after diabetic retinopathy.^1^ Although RVO is frequently seen in people older than 65 years, it is an important reason of vision loss that may also affect the young people. It is more common in men. Retinal vein occlusions are classified as central, hemi-central and branch occlusions according to the level of occlusion.^2^ While the occlusion is at disc level in central and hemi-central vein occlusions, in branch retinal vein occlusion (BRVO) it is at arteriovenous crossing point.^3^ The prevalences of BRVO and central retinal vein occlusion (CRVO) are about 0.6% and 0.1%, respectively.^4^

The most common risk factors of RVO are systemic vascular diseases (diabetes, hypertension, hyperlipidemia) and elder age (> 65 years).^2^,^3^ RVO is affected by different factors including the use of drugs (Oral Contraseptives (OCC), hormone treatments), various rheumatic diseases, infections, deficiency of protein C, protein S and antithrombin that cause susceptibility to thrombophilia, antiphospholipid antibodies, hyperhomocysteinaemia, genetic mutations (Factor V Leiden mutation, MTHFR (methylenetetrahydrofolate reductase) mutation, prothrombin gene mutation) and hyperviscosity syndromes (polycythemia rubra, leukemia, lymphoma).^5^ These factors can play an important role especially in young RVO patients. Schockman et al.^6^ reported that factor V Leiden and / or prothrombin mutation (9%), low protein S levels (9%) and elevated homocysteine levels (23%) were the predisposing risk factors in RVO patients.

In young patients, determining the factors that may cause predisposition to RVO may affect the progress of the disease positively by decreasing the recurrence of it or its potential occurrences in the other eye. Accordingly, the potential etiological factors that may cause RVO in young patients should undergo careful systemic examination and detailed laboratory analysis.

## METHODS

The study was conducted at Ege University Medicine Faculty Department of Ophthalmology. The clinical records of patients who were diagnosed with RVO under the age of 50 between January 2014 and March 2018 were analyzed retrospectively. Forty patients comprised the study. The study was carried out by getting the approval of Ege University, Faculty of Medicine, Clinical Researches Ethical Committee and being in conformity with Helsinki Declaration and written informed consent forms were taken from all patients. The clinical findings, fundus photos and medical records of these patients were reviewed by two different retina experts. Based on these data, CRVO was identified as optic nerve head swelling, retinal hemorrhages in all four quadrants of the fundus with a dilated and tortuous retinal venous system. BRVO was identified as the presence of retinal hemorrhages and exudates after any arteriovenous crossing point with dilated and tortuous retinal veins in the said quadrant. The diagnoses were verified with fluorescein angiography in the consideration of venous filling defects and extended venous filling time.

Together with the demographic data of the patients such as age and gender, ophthalmological data such as the best corrected visual acuity (BCVA) evaluated by logarithm of the Minimum Angle of Resolution (logMAR), Intraocular Pressure (IOP) measured by Goldmann applanation tonometry, refractive error, type of vein occlusion and glaucoma history were recorded. Fundus photos, optic coherence tomography images and fluorescein angiography data of all patients were evaluated and recorded.

Besides these, the examinations performed for systemic diseases such as diabetes, hypertension, Behçet’s disease and use of medicines (OCC etc.) were also recorded. Smokers were excluded from the study because the amount of smoking may vary widely among individuals. All patients were examined further in terms of mainly systemic hypertension, diabetes, hyperlipidemia and cardiovascular diseases and other thrombophilic features (hyperhomocysteinaemia, MTHFR mutation, factor V Leiden mutation, deficiency of protein C, deficiency of protein S, protein C resistance, prothrombin gene mutation, antiphospholipid syndrome) that may lead to RVO, hyperviscosity syndromes (leucemia, lymphoma, polycythemia, multipl myeloma) and other pathologies that may cause vasculitis (Behçet’s disease, sarcoidosis, syphilis, systemic lupus erythematosus). Additionally, all of them were investigated with the consultation of relevant departments.

## RESULTS

Twenty-two (55%) patients were male and 18 (45%) patients were female. The mean age was 41.6 ± 10.01 (range 22 – 50 years). While CRVO was diagnosed in 18 (45%) patients, BRVO was diagnosed in 22 (55%) patients (Table-I).

**Table I:** Demographic data of patients.

	CRVO (n=18), n(45%)	BRVO (n=22), n (55%)
***Gender***		
Male	9 (50%)	13 (59.1%)
Female	9 (50%)	9 (40.9%)
***Age***		
< 30	7 (38.9%)	3 (13.6%)
30 - 40	3 (16.7%)	5 (22.7%)
41 - 50	8 (44.4%)	14 (63.6%)
Mean	35.4 ± 10.8	42.00 ± 9.4

CRVO, Central Retinal Vein Occlusion; BRVO, Branch Retinal Vein Occlusion.

The mean BCVA was 0.76 ± 0.64 logMAR (0 - 2). This value was 0.52 ± 0.28 logMAR (0.05-0.7) in patients with BRVO and as 0.89 ± 0.67 logMAR (0-2) in patients with CRVO.

Mean IOP was 16.8 ± 5.47mmHg (10 - 28) and there was one patient with glaucoma history. Six (15%) patients were hypermetropic and 11 (27.5%) patients were myopic. There was no refractive error in 23 (57.5%) patients. Other ocular causes of RVO like uveitis were not detected.

In the systemic etiological researches, there was an OCC usage history in two (5%) patients (one CRVO, one BRVO). While two (9%) of the BRVO patients had diabetes and eight (36.3%) had hypertension, two (11.1%) of the CRVO patients had diabetes and four (22.2%) had hypertension. In two of these patients, BRVO was found as the first finding for Behçet’s disease. The distribution of patients with respect to etiology and BRVO/CRVO is given in Table-II. The MTHFR gene mutation was in four (36.3%) patients homozygous and in seven (63.7%) patients heterozygous. The prothrombin gene mutation was in two (50%) patients homozygous and in two (50%) patients heterozygous. All (100%) of the factor V Leiden gene mutations were heterozygous. Eleven (27.5%) patients had multiple etiological factors. The distribution of the multiple etiological factors is given in Table-III.

**Table II:** Distribution of Etiological Factors.

	CRVO (n=18)	BRVO (n=22)	TOTAL (n=40)(%)
***Etiological Factor***			
Use of OCC	1	1	2 (5%)
Hyperhomocysteinaemia	8	7	15 (37.5%)
Behçet’s Disease	-	3	3 (7.5%)
MTHFR gene mutation	5	6	11 (27.5%)
Prothrombin mutation	3	1	4 (10%)
Factor V Leiden mutation	2	3	5 (12.5)
Diabetes mellitus	2	2	4 (10%)
Hypertension	4	8	12 (30%)
Glaucoma	1	-	1 (2.5%)

CRVO, Central Retinal Vein Occlusion; BRVO, Branch Retinal Vein Occlusion; OCC, Oral Contraceptives; MTHFR, Methylenetetrahydrofolate reductase.

**Table III:** Distribution of Multiple Etiological Factors.

Patients/Etiological Factors	1	2	3	4	5	6	7	8	9	10	11
Behçet’s disease	X										
Diabetes and/or Hypertension		X	X	X	X						
Hyperhomocystenemia		X	X	X	X			X	X	X	
Prothrombin mutation					X		X				X
MTHFR gene mutation	X					X	X	X	X	X	X
Factor V Leiden mutation	X					X	X				

All of these patients were brought under control in the relevant units for monitoring and treatment of these pathologies. The data obtained in this study for the management of young RVO cases are discussed below and compared with the literature.

## DISCUSSION

Retinal vein occlusion is rising by the increase of systemic vascular risk factors in elderly age and especially systemic hypertension is the vascular pathology that is most associated with RVO in this group.^2^ However distribution of risk factors in RVO patients under the age of 50 years is varying significantly and should be evaluated in a broader perspective. This study presents the etiological research results in terms of RVO in young RVO cases who were diagnosed before the age of 50 years.

In the pathogenesis of retinal vein occlusion, Virchow’s triad defined as hemodynamic changes (venous stasis), degenerative changes in vein walls and increased thrombosis trend is important.^2^ The most important factors in retinal vein occlusion are elder age and systemic vascular diseases. These risk factors come into prominence in RVO cases above the age of 65. Hayreh et al.^7^ determined that 51% of RVO cases were elder than 65 years.

In the development of retinal vein occlusion another risk factor is gender. As in other vascular diseases, male patients are more susceptible to RVO. In their studies consisting of 103 cases, Fong et al.^8^ reported that 64% of the patients were male. Also in our study, 55% of the patients were male and 45% of them were female. However, there are studies which indicated that RVO is more common in female.^9^

Other important systemic risk factors for RVO are hypertension, hyperlipidemia and diabetes mellitus. According to data obtained from the studies, RVO is related with 48% hypertension, 20% hyperlipidemia and 5% diabetes mellitus.^2^ In many previous studies, it was reported that RVO is especially seen in median and elder ages and related with hypertension, diabetes, hyperlipidemia, cardiovascular diseases and open-angle glaucoma.^2^,^5^,^10^-^12^ In our study, hypertension was determined in 12 (30%) patients, diabetes in four (10%) and glaucoma in one (2.5%) patient. These results were compatible with the literature.

Use of OCC is reported as an important risk factor for vascular occlusions especially in young female patients.^13^,^14^ Gynecologists generally suggest OCC usage for birth control. Additionally, since OCC is used for the regulation of menstrual cycle for in vitro fertilization, use of OCC in young age group significantly increased today. Although it was not clearly identified how use of OCC leads to RVO, there are many studies suggesting that it increases RVO risk.^13^,^15^,^16^ In our study, there were two (5%) patients who were using OCC but did not have any additionally risk factors in terms of RVO.

Homocysteine is an amino acid that acts in methionine metabolism and which contains sulfur. Homocysteine converts into methionine by means of MTHFR enzyme. The mutations occurring in the gene that encodes this enzyme cause hyperhomocysteinaemia and lead to an increase in the risk of RVO and other neurological and cardiovascular diseases.^17^ While the heterozygous mutations in this gene are decreasing enzyme activity by 30%, homozygous mutations decrease this by 60%.^18^ The relation between hyperhomocysteinaemia and RVO was reported in various studies. Li et al.^19^ reported that there is a relation between plasma homocystein and RVO. However, they could not find a relation between MTHFR gene mutation and RVO. Starting from this point, it was suggested that the genetic factors alone are not enough to increase homocysteine levels and plasma homocysteine levels are influenced by age, gender, folate intake, smoking, vitamin B levels, systemic vascular diseases and use of anti-hypertensive medicines.^20^ In the literature, there are reported rates varying between 3-19% in terms of MTHFR gene mutation and hyperhomocysteinaemia relation.^11^,^21^ In our study, while there was hyperhomocysteinaemia in 15 (37.5%) patients, MTHFR gene mutation was diagnosed in 11 (27.5%) patients.

Different studies have reported that factor V Leiden and prothrombin gene mutations are effective in the development of RVO.^6^ Among these, factor V Leiden mutation is the genetic factor that causes thrombosis most frequently.^22^ This mutation is seen in white race in the frequency of 5%.^22^ When this mutation occurs, protein C becomes resistance to enzymatic degradation and causes hypercoagulability. It was reported that this mutation increases the risk of thrombosis 3-8 folds in individuals that are heterozygous and 80 folds in ones that are homozygous. It was seen that factor V Leiden mutation existed in 20% of patients with systemic vein thrombosis.^22^ Caprini et al. reported that there was factor V Leiden mutation in 17% of patients with deep vein thrombosis history and 27.3% of the patients with pulmonary embolism.^22^-^24^ Turello et al. detected heterozygous factor V Leiden mutation in 8.2% of the patients with RVO.^25^ In our study, while heterozygous factor V Leiden mutation was detected in five (12.5%) patients, homozygous mutation was not detected. The reason of such diversity in the rates could be the fact that the studies had been implemented on different races.

Prothrombin gene mutation may increase the thrombosis risk by three folds by causing too much prothrombin production. It is the most frequent mutation seen after Factor V Leiden mutation. It was reported that the prothrombin gene mutation was seen in 5.5% of patients with thrombosis and 1.2% in normal individuals.^22^ Caprini et al. reported that this rate was 17% in patients with vein thrombosis history and 18.2% in patients with pulmonary embolism history.^22^ In a study that was carried out without making a distinction of young or elder age RVO, prothrombin gene mutation rate was determined as 4.2%.^25^ Despite this fact, in our study such rate was found as 10% (four patients). This result is supporting that genetic factors in terms of etiology are more effective in young patients with RVO.

Although retinal vein occlusion is frequently seen in patients above 65 years, it is a retinal vascular pathology that can be seen in early ages and that threatens the vision. While there are limited number of risk factors in elder ages regarding this pathology, risk factors in young patients are presenting a broader distribution. Starting from this point, etiological researches in a young RVO case should be considered more broadly including genetic tests. The positive results that can be obtained in terms of risk factors may ensure the treatment of these factors, which may cause thrombosis predisposition and may help to prevent development of new RVOs and potential vascular problems in other organs.

### Authors’ Contribution

**SN:** Conceived, designed and manuscript writing.

**CD:** Did data collection and editing of manuscript.

**CA:** Did review and final approval of manuscript.

**JM:** Did final approval of manuscript.
